# Supplementary material for: Patterns of Intron Gain and Loss in Fungi
Source: PLoS Biol. 2004 Nov 30;2(12):e422. doi: 10.1371/journal.pbio.0020422 (PMC532390; doi:10.1371/journal.pbio.0020422)
Supplement: Table S1 — Also available at http://genes.mit.edu/NielsenEtAl/. (4.3 MB ZIP). [file pbio.0020422.st001.zip › NielsenEtAl/html/209.html]

AN4939.1.NCU07286.1.MG00274.1.FG02758.1


```
 CLUSTAL W (1.82) Multiple Sequence Alignments - Introns Inserted


Sequence 1: MG00274.1	188 aa
Sequence 2: FG02758.1	171 aa
Sequence 3: NCU07286.1	173 aa
Sequence 4: AN4939.1	167 aa
Alignment Length: 190 aa
Number Identitical Residues: 82 aa
Alignment Score (without introns) 3988


MG00274.1 	MDYV~EQRVAQEAKKLAEQPAT~EYAFTTAAPGLIPR2A~SFITPLNIILLAVVAYTLYA
NCU07286.1	MDYV~EN-------RMAQEAAK~QTG---------NA~E2SFVTPLNLVLLGMLLYTAYS
FG02758.1 	MDHV~DS-------HLAHQASQ~E------------N2V~SFFTPLNLVLICAVLYTTYA
AN4939.1  	MSAE1GT---------APQAEV2A-------------~-~SIATPINLILFSAFAFLVYT
          	*.              * :.                    *: **:*::*:  . :  *:

MG00274.1 	VFRPAPPPPMPREAPATVFRTFTPPTLIHYNGQDGKPVYLAVRGRVFDVTAGRNFYGP~G
NCU07286.1	WLRPHTPATIPKEEPAVVFKTFTPRTLLPYNGEKDMPVYLAVRGRVFDVTRGRNFYGP~G
FG02758.1 	LMRSSPPPSLPRDTPSTVFRTYNPHTLLPFNGEEGRPVYLAVRGRVFDVSPGRNFYGP~G
AN4939.1  	QFRPKPPAVLPKAPAPVVFRTFTPKTLLPFNGENDKPVYLAVRGRVFDVTPGRNFYGP0G
          	 :*. .*. :*:  ...**:*:.* **: :**:.. *************: ******* *

MG00274.1 	GPYENFAGRDASRGLAHHSFDQDMLTLDLNGPLDKLDDLGPDELAALRDWEERFESKYLV
NCU07286.1	GPYANFAGRDASRGLACGSFDEEMLTKDLDGPLDTLSDLDKDQLEALQGWEERFLEKYLV
FG02758.1 	GPYSNFAGRDASRGLACGSFDEDMLTKDLNAPLDKLEGLDEEQMDALRGWEERFLEKYNV
AN4939.1  	GPYENFAGRDASRGLAFQSFDKEMLTEDLSGPLDDLKDLNADQLENLQSWEERFLEKYLV
          	*** ************  ***::*** **..*** *..*. :::  *:.***** .** *

MG00274.1 	VGRLVAVGDEEKK--
NCU07286.1	VGKLVAVGDEDKAE-
FG02758.1 	VGKLVSMQDYEAQKA
AN4939.1  	VGKLVAEGDPEAPKS
          	**:**:  * :  .:
```
